# The MARCH Family E3 Ubiquitin Ligase K5 Alters Monocyte Metabolism and Proliferation through Receptor Tyrosine Kinase Modulation

**DOI:** 10.1371/journal.ppat.1001331

**Published:** 2011-04-07

**Authors:** Roshan Karki, Sabine M. Lang, Robert E. Means

**Affiliations:** Department of Pathology, Yale University School of Medicine, New Haven, Connecticut, United States of America; University of North Carolina at Chapel Hill, United States of America

## Abstract

Kaposi's sarcoma (KS) lesions are complex mixtures of KS-associated herpesvirus (KSHV)-infected spindle and inflammatory cells. In order to survive the host immune responses, KSHV encodes a number of immunomodulatory proteins, including the E3 ubiquitin ligase K5. In exploring the role of this viral protein in monocytes, we made the surprising discovery that in addition to a potential role in down regulation of immune responses, K5 also contributes to increased proliferation and alters cellular metabolism. This ubiquitin ligase increases aerobic glycolysis and lactate production through modulation of cellular growth factor-binding receptor tyrosine kinase endocytosis, increasing the sensitivity of cells to autocrine and paracrine factors. This leads to an altered pattern of cellular phosphorylation, increases in Akt activation and a longer duration of Erk1/2 phosphorylation. Overall, we believe this to be the first report of a virally-encoded ubiquitin ligase potentially contributing to oncogenesis through alterations in growth factor signaling cascades and opens a new avenue of research in K5 biology.

## Author Summary

Tumor viruses have proven to be valuable tools for dissecting the molecular mechanisms of transformation and cancer progression. Kaposi's sarcoma-associated herpesvirus (KSHV) infection is essential in driving at least three different neoplasias, including Kaposi's sarcoma (KS). Our understanding, however, of the molecular mechanism of KSHV-driven tumor progression is still limited and requires further examination. In this manuscript we demonstrate that the K5 E3 ubiquitin ligase of KSHV is able to alter monocyte metabolism, driving increased glucose consumption and lactate production, hallmarks of virtually every cancer. It is able to accomplish this through a modulation of selected receptor tyrosine kinases, whose normal role is to bind pro-growth factors. Indeed, this alteration in metabolism is coupled with increases in monocyte proliferation. Our study provides insights into the mechanisms of KSHV-driven oncogenesis, as well as a new tool for exploring the link between metabolism and cancer.

## Introduction

Tumor progression is a convoluted process that involves changes in tumor-initiating cells and the surrounding stroma. Increases in glucose uptake and lactate production are salient features of about 90% of all cancer cells and are routinely used in the clinical setting to identify tumor cells using PET and HMR spectroscopy [1], [2]. Stromal cells, thought to be important in the maintenance and metastasis of tumors, also exhibit similar changes in metabolic profiles driven by factors released from the transformed cells [1], [3]. Understanding and dissecting the role of stromal cells in metastatic progression is made difficult by the fact that changes in these cells are not due to genetic lesions, but are a product of the surrounding microenvironment. Historically, tumor viruses have proven to be valuable tools for dissecting the molecular mechanisms of transformation since these pathogens, by definition, encode at least the minimal requirements needed for tumorigenesis in their host. It is clear from almost two decades of research that products encoded by Kaposi's sarcoma-associated herpesvirus (KSHV) are essential in driving at least three different neoplasias, Kaposi's sarcoma (KS), primary effusion lymphoma (PEL) and plasmablastic multicentric Castleman's disease (MCD) [4]-[6]. Our understanding, however, of the mechanism of KSHV-driven tumor progression is still limited and requires further examination.

KS lesions are histologically complex, comprised of KSHV-infected endothelial cells, but also infiltrating inflammatory cells. The role of these cells in the pathology of the disease or in the viral life cycle is somewhat unclear. As with a variety of tumors resulting from genetic lesions, monocytes and monocyte-derived cells within KS lesions could play a critical role in tumor progression, releasing a variety of cytokines that promote expansion of the surrounding latently infected cells, while suppressing or skewing the anti-tumor immune response [7]-[9]. Indeed, KSHV-driven neoplasias are known to be dependent on a variety of both virally- and host-encoded cytokines and growth factors [10]-[13]. A variety of publications have described alterations in monocyte-lineage cell function following either KSHV infection or in response to virally encoded products. For example, the viral OX2 homologue has been shown to alter cytokine release from macrophages both *in cis* and *in trans*, increasing IL-1ß, IL-6 and TNF-α, also suppressing IFN-γ stimulated immune responses [14], [15]. Similar up regulation of IL-6 and IL-10 expression was seen following expression of two KSHV-encoded microRNAs in monocytes [16]. Infection of the THP-1 human monocyte cell line has been shown to alter TLR3 expression and function, mediating increased release of CXCL10 and IFN-β1 [17]. These lines of data open the door for direct infection of monocytes playing a role in tumor progression, further supported by the ability of KSHV to utilize DC-SIGN, a molecule expressed on monocyte-derived cells and activated B cells, as a co-receptor to increase infectivity [18]-[20].

A number of both lytic and latent KSHV encoded gene products are believed to play a role in KS pathogenesis. The KSHV latency-associated proteins are readily detectable in KS tumors, however, none of these products has been shown to be sufficient for inducing sarcomagenesis in mouse models [21], [22]. On the other hand, lytic gene products can transform endothelial cells *in vitro* to various efficiencies and murine KSHV models show vGPCR to be a major oncogene [21], [23]. However, only a subset of cells within KS tumors, not the predominant spindle-shaped endothelial cells, display a lytic pattern of gene expression confounding the contribution of KSHV lytic genes in KS pathogenesis [24], [25]. By definition, cells expressing the lytic gene program are fated to die, giving rise to hypothesis that a low proportion of lytically-infected cells in the tumor are providing paracrine factors to promote tumorigenesis.

The K5 protein of KSHV (also termed modulator of immune responses 2 (MIR 2)) is the prototypical member of the membrane-associated, RING-CH containing (MARCH) E3 ubiquitin ligase family. Work from our lab and from many others have shown that this protein is able to target a variety of immunomodulatory proteins for down regulation, presumably leading to reduced immune responses against virally-infected cells (reviewed in [26]). While K5 has been characterized as a lytic protein, its expression is also driven by cellular Notch signaling, potentially allowing for expression independent of the full lytic cycle [27], [28]. A more recent report looking at the epigenetic status of the KSHV genome has reported a large number of activating histone modifications and a paucity of inhibitory marks in the K5 gene loci in latently infected cells, raising questions about its definition as a lytic gene product [29]. Finally, immuno-histochemical studies have shown that K5 is expressed in KS tumors, as well as in PEL and MCD together raising the intriguing possibility of a role for K5 in KSHV mediated oncogenesis [30].

Given a potential role for monocyte-derived cells in the pathogenesis of KSHV-driven neoplasias and the ability of the KSHV K5 protein to regulate the anti-viral immune responses, we undertook a study of the ability of this immune evasion molecule to alter monocyte functionality. To our surprise, we observed that in addition to potentially altering anti-viral immune responses, the K5 protein is able to alter monocyte metabolism and replication rate. K5-mediated alterations potentially contribute to tumorigenesis by altering the surrounding microenvironment through induction of aerobic glycolysis with release of lactate and increasing sensitivity of tumor cells to paracrine factors. Mechanistically, this occurs through targeting and re-localization of a subset of growth factor-binding receptor tyrosine kinases, enhancing pro-proliferation and survival signaling. We believe that this report represents the first example of a virally-encoded E3 ubiquitin ligase acting as a transforming factor.

## Results

### KSHV K5 alters monocyte proliferation

**Figure 1:**
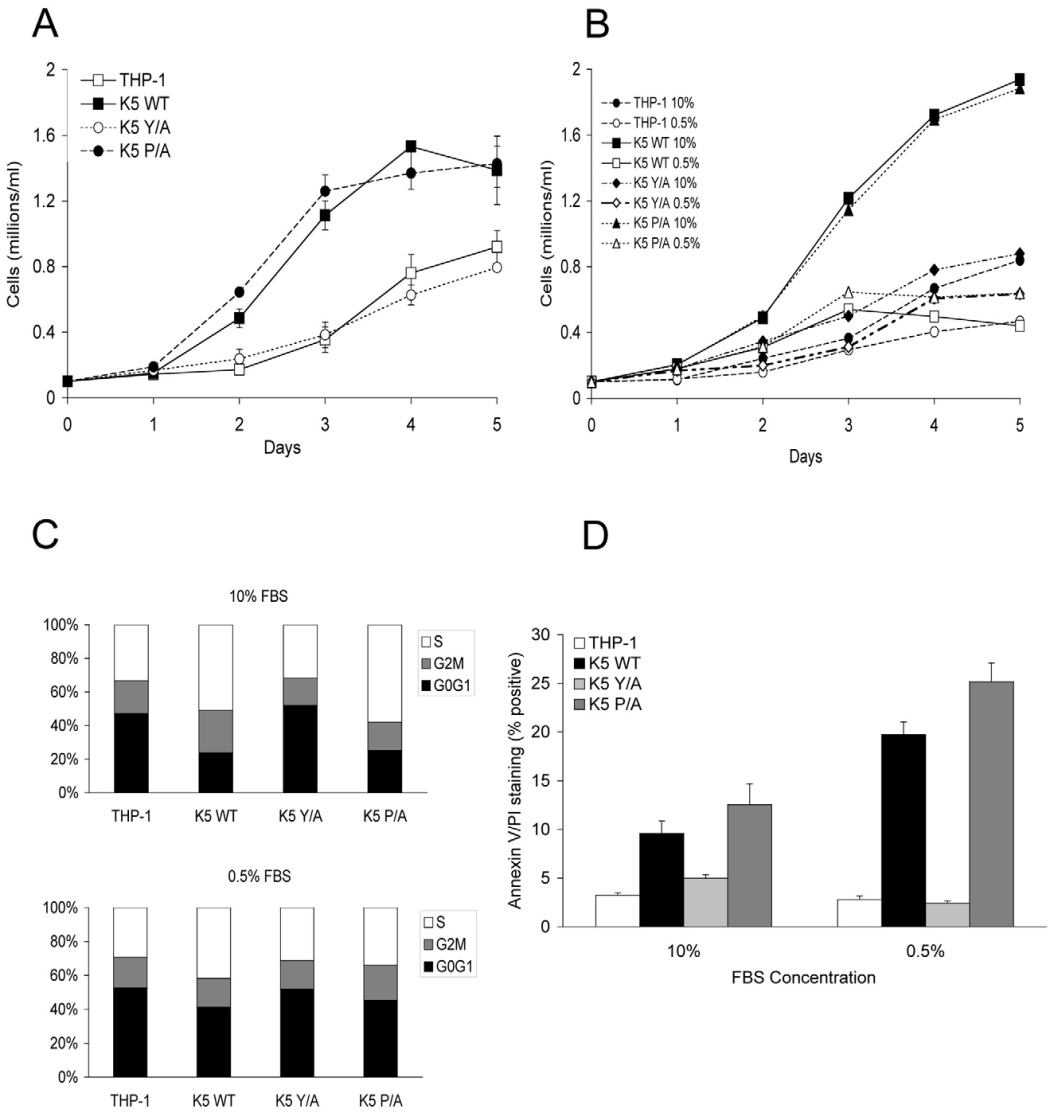
THP-1 cells stably expressing wild-type K5 have increased growth rate, which is serum dependent. THP-1 cell lines were seeded at 0.1 x 10^6^ cells/ml in RPMI media supplemented with (**A**) 10% FBS or (**B**) 5% and 0.5% FBS, as indicated, in triplicates. Viable cell numbers and averages were determined for each day. (**C**) Equal numbers of indicated THP-1 cell line in medium with either 10% FBS (top panel) or 0.5% FBS (bottom panel), were examined for cell cycle profile, as described in the Methods, at three days post-seeding. Results are representative of three independent experiments. (**D**) Each of the indicated THP-1 cells was seeded in triplicate at 0.1 x 10^6^ cells/ml in medium containing the indicated serum concentration. Five days post-seeding cells were examined for apoptosis. The percentage of cells doubly stained for Annexin V and propidium iodide are indicated.

To investigate a potential role for K5 in altering monocyte functionality, the human monocyte cell line THP-1 was transduced to stably express empty vector or WT (wild-type) K5. Unexpectedly, following antibiotic selection, cells expressing WT K5 had a markedly decreased doubling time (∼15 hours vs. ∼28 hours) and achieved higher culture densities than vector-expressing THP-1 cells. To ensure this was not an artifact of selection, retroviral transduction of cells was repeated, using additional viruses encoding mutants of K5 that alter the ability of the protein to down regulate immunomodulatory proteins [31]. Once again, cells expressing WT K5 had a significantly increased rate of growth as compared with vector-transduced cells (Fig. 1A). Additionally, this ability to increase cellular replication rate was maintained by K5 expression constructs containing a mutation of the proline-rich, potential SH3-binding domain (K5 P/A) or the downstream acidic residues (K5 DE12) (Fig. 1A and data not shown). However, three other constructs, K5 Y/A, containing a mutation in the tyrosine-based endocytosis motif and two constructs lacking E3 ubiquitin ligase activity, mZn and W47A, did not significantly alter the growth rate of THP-1 monocytes (Fig. 1A and data not shown).

As a first exploration of the mechanisms underlying this increased proliferation, vector expressing- and WT K5-expressing THP-1 cells were tested for serum dependency. At low serum concentrations (0.5% FBS), there was minimal change in the growth rate of vector cells as compared with growth in 10% FBS, while there was a dramatic reduction in proliferation of the WT K5-expressing cells (Fig. 1B). Examination of the cell cycle profile of these cells revealed that in the presence of high serum concentrations approximately 15% more WT K5 cells were in S-phase, with a significant decrease in cells in G0/G1, as compared to vector-expressing cells (Fig. 1C). This same increase in S-phase cells was seen for the K5 P/A-expressing cells, while the cell cycle profile of those expressing the K5 Y/A mutant resembled the THP-1 vector cells. Switching the WT K5 or K5 P/A cells to low serum resulted in a decrease in S-phase cells, with concomitant increase in cells in G0/G1, such that they resembled vector-expressing cells. Co-staining of cells with Annexin V and propidium iodide revealed that less than 5% of vector-THP-1 or K5 Y/A-expressing cells stained positive for apoptosis in the different serum concentrations. However, in WT K5 and K5 P/A cells, the percentage of cells undergoing apoptosis increased from ∼10% in high serum conditions to greater than 20% of cells under low serum conditions (Fig. 1D). Intriguingly, in medium containing 10% charcoal-stripped FBS, WT K5 cells still maintained a high proliferation rate, while vector-THP-1 cells exhibited a significantly decreased proliferation rate (Fig. S1A). Together, this data suggests that K5 might be acting to increase monocyte responsiveness to serum-derived growth factors.

### K5 expressing THP-1 cells have altered metabolism

**Figure 2:**
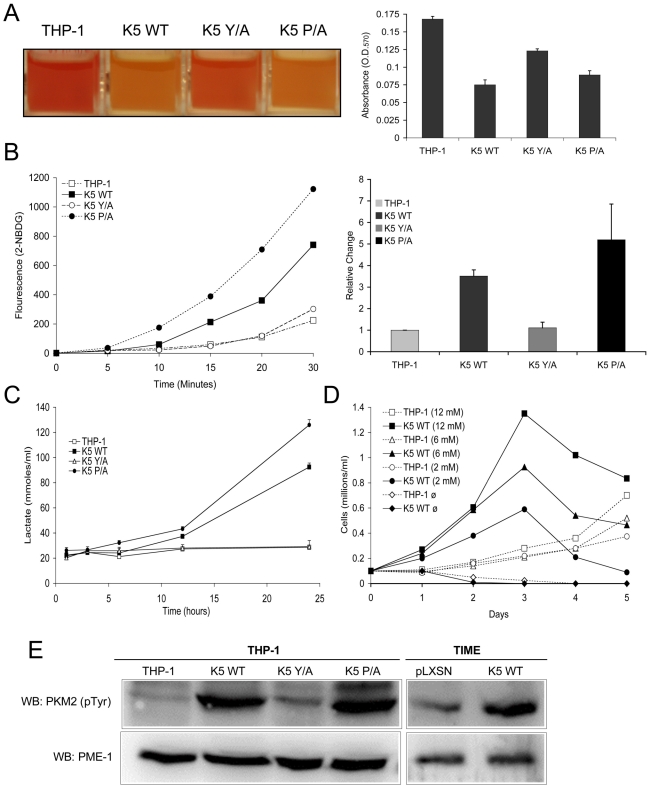
THP-1 and TIME cells stably expressing wild-type K5 have an altered metabolism. (**A**) Medium from equal numbers of the indicated cells were examined at 3 days post-seeding both visually (left panel) and for absorbance at 570 nm (O.D._570_) (right panel) (**B**) Equal numbers of each THP-1 cell line were starved for 3 hours and incubated in glucose free DMEM medium with a fluorescent glucose analog (2-NBDG) (0.1 mM) for a period of 30 minutes. Live cells were gated and the fluorescence intensity assessed by flow cytometry at the indicated time points. A representative experiment of analog uptake (left panel) and the quantification of fold change after 30 min. with standard deviations (right panel) are shown. (**C**) Cells were seeded in triplicate at 0.1 x 10^6^ cells/ml and supernatant was taken at the indicated time points and subjected to colorimetric measurement of lactate concentration. (**D**) Cells were seeded at 0.1 x 10^6^ cells/ml in glucose-free DMEM media supplemented with different concentrations of glucose (12 mM, 6 mM, 2 mM or 0 mM). Viable cell numbers were determined daily. Data are representative of three experiments. (**E**) Normalized whole cell lysate of each of the indicated THP-1 and TIME cell lines were subject to western blot (WB) using an anti-phospho-PKM2 (Tyr 105) antibody and re-probed with anti-PME-1 antibodies.

During growth rate analysis, we also noticed that any of the THP-1 lines expressing a K5 construct which decreased doubling time also acidified the growth medium at a more rapid rate, independent of cell number, as compared to vector-expressing THP-1 cells. Over a one day period the medium containing vector-THP-1 or K5 Y/A expressing THP-1 cells decreased in pH from 7.8 to 7.0, while the medium from K5 WT or K5 P/A-expressing cells dropped to 6.5. This drop in pH was visible as a change in indicator color in the medium which could also be measured as a drop in optical density at 570 n.m. (Fig. 2A). This led us to question whether this rapid change in culture pH due to a metabolic shift of cells to increased aerobic glycolysis, a process that occurs in a virtual plurality of tumors and is dubbed the ÒWarburg EffectÓ (reviewed in [32]). Equal numbers of WT K5-, K5 Y/A-, K5 P/A and vector-expressing THP-1 cells were incubated with the fluorescent glucose analog 2-NBDG and uptake was measured over a period of thirty minutes. There was a greater than four fold increase in the uptake of glucose in K5-WT and K5 P/A-expressing THP-1 cells when compared to the parental cells (Fig. 2B). Lactate secretion into the media was followed over a longer, 24-hour period. Equal numbers of vector-, K5 WT-, K5 Y/A and K5 P/A-expressing THP-1 cells were seeded into culture vessels, samples were taken at various time points, cells enumerated and supernatant was subjected to colorimetric measurement of lactate concentration. After normalization for cell number, the rate of lactate production over the 24 hour period for K5-expressing THP-1 cells was 3.80 mmoles/hour/1*10^5^ cells, almost ten fold more than in vector-expressing THP-1 at 0.41 mmoles/hour/1*10^5^ cells (Fig. 2C). The K5 P/A- and K5 Y/A-expressing cells behaved similarly to K5 WT- and vector-expressing THP-1 cells, respectively.

To determine if this ability of the K5 protein to alter cellular metabolism and increase lactate production was limited to monocytes, we established K5-expressing telomerase-immortalized microvascular endothelial cells (TIME). TIME cells have been previously used by a number of groups to model KSHV infection of endothelial cells in the KS tumors [33], [34]. As in the THP-1 cells, K5-expression was very low and observed only following protein immunoprecipitation however, we were able to demonstrate down regulation of MHC I, indicating that K5 is functional in TIME cells (data not shown). We did not observe a decrease in doubling time, but we did observe an approximately 2.3 fold increased lactate production in medium containing high concentrations of growth factors, as compared with TIME cells transduced with vector alone (data not shown). This increase in lactate production was amplified when cells were switched to DMEM supplemented with 10% serum only with TIME K5 WT cells producing almost 5 fold more. Finally, we also examined lactate production in KSHV-infected TIME cells. The published literature indicates that at short-time points following infection there are increases in lactate production [35]. We did not observe such an increase at the longer time post-infection (greater than 3 weeks) points examined in our studies (data not shown).

Another feature of many cancer cells that have shifted their metabolism to aerobic glycolysis is an increased dependence on glucose for growth and an up regulation of glucose transporters [36]. To test if WT K5-expressing THP-1 cells are addicted to glucose for their growth, equal numbers of cells were seeded in complete medium with different concentrations of glucose (0-12mM). While vector-expressing THP-1 failed to proliferate in glucose-free media, their growth rate was essentially unchanged under low versus high glucose concentrations (Fig. 2D). In contrast, the proliferation rate of the K5-expressing cells was directly proportional to the concentration of glucose in the media. We observed similar results when varying the glutamine concentration (Fig. S1B). RT-PCR array was used to examine levels of glucose transporter message in K5- versus vector-expressing cells. Overall levels of GLUT4 (SLC2A4) message increased almost 700 fold, while more modest increases in both GLUT1 (SLC2A1) and GLUT3 (SLC2A3) were observed (Table S1).

As a final measurement of metabolic shift induced by K5 expression, we measured the relative amounts of phosphorylated pyruvate kinase M2 isozyme. Phosphorylation of the M2 form decreases the ability of the enzyme to form homo-tetramers, negatively impacting its ability to convert phosphoenolpyruvate to pyruvate, promoting aerobic glycolysis [37], [38]. Western blot (WB) analysis with a phospho-specific PKM2 antibody revealed a dramatic increase in reactive protein in both K5 WT- and K5 P/A-expressing THP-1, as compared to either vector- or K5 Y/A-expressing cells (Fig. 2E). An increase in the phosphorylated PKM2 was also seen in TIME K5 cells as compared to TIME cells expressing vector only (Fig. 2E).

### K5 induces changes in HIF-1α and Akt activation

**Figure 3:**
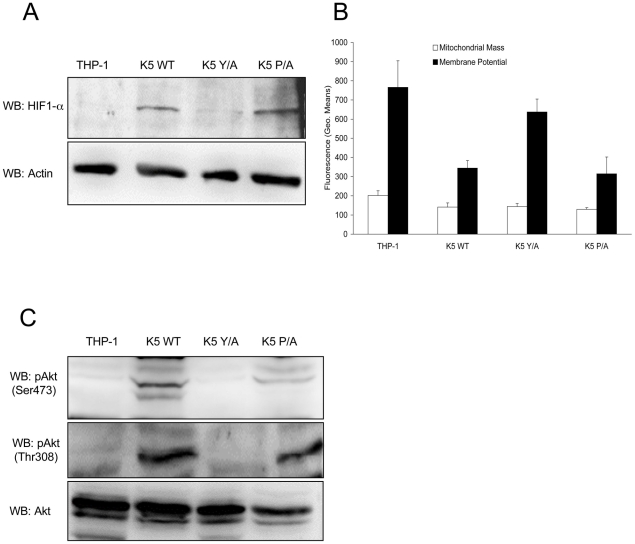
K5 expressing cells have other features of the Warburg Effect. (**A**) Normalized whole cell lysate of each of the indicated THP-1 cell lines were subject to western blot (WB) using an anti-HIF-1α antibody (top panel) and re-probed with anti-ß-actin antibodies (bottom panel). (**B**) Equal numbers of the indicated cell lines were stained with mitotracker green (Mitochondrial Mass) or mitotracker red (Membrane Potential) and subjected to flow cytometric analysis. (**C**) Normalized whole cell lysates were subjected to western blot (WB) using anti-phospho-Akt (Ser473) (top panel), anti-phospho-Akt (Thr308) (middle panel) or total Akt (bottom panel) antibodies. Data for all panels are representative of three independent experiments.

One of the fundamental contributors to the Warburg Effect has been suggested to be the HIF-1α protein, the master regulator of oxygen homeostasis in cells [39], [40]. Although induced by hypoxia in the low oxygen tension tumor microenvironment, it can also be stabilized under normoxic conditions by multiple pathways involving increased glycolysis and lactate production, Akt pathway activation, or loss of the PTEN tumor suppressor [41]-[44]. WB analysis of normalized whole cell lysates (WCL) from THP-1 lines cells grown under normoxic conditions demonstrated readily detectable HIF-1α protein in both WT K5- and K5 P/A-expressing THP-1 cells, while HIF-1α protein was almost undetectable in vector- and K5 Y/A expressing cells (Fig. 3A).

There is evidence in the literature that the HIF-1α protein can negatively regulate mitochondrial biogenesis, contributing to the decreased respiration under aerobic conditions seen for many tumor lines [45]. Equal numbers of vector-, WT K5-, K5 Y/A- and K5 P/A-expressing THP-1 cells were subject to flow cytometry analysis using the fluorescent stains, Mitotracker Green (mass) and Mitotracker Red (membrane potential). In contrast to the expected result, quantification of the relative levels of staining in each cell line indicates that mitochondrial mass was preserved, but membrane potential in the WT K5- and K5 P/A-expressing THP-1, which also demonstrated increased HIF-1α protein levels, were two fold lower as compared to vector- and K5 Y/A-expressing cells (Fig. 3B). Interestingly, WT K5-expressing THP-1 maintains an equal or slightly increased oxygen consumption rate as compared to vector-expressing THP-1 (data not shown).

HIF-1α protein stabilization has also been implicated in activating Akt under normoxic conditions [46], [47]. Furthermore, Akt is not only the most activated serine/threonine kinase in myriad of cancers, but it has also been shown to be an important inducer of the Warburg effect by stimulating aerobic glucose metabolism through increased expression of GLUT receptors [36], [48], [49]. Normalized WCL from the THP-1 lines were subjected to WB, probing with p-Akt (Ser 473) and p-Akt (Thr 308) antibodies. There was comparatively increased phosphorylation of Akt at both sites in WT K5- and K5 P/A-expressing THP-1 cells, while total Akt levels were essentially equal in all cells (Fig. 3C).

### K5-expressing THP-1 have altered total phosphorylation levels

Akt is activated in a phosphoinositide 3-kinase (PI3K)-dependent manner that is downstream of growth factor receptor tyrosine kinases (RTKs) [50]. Since WT K5-expressing THP-1 cells show robust activation of Akt and serum dependency, we decided to explore the activation of RTKs and their contribution to subsequent downstream signaling through the PI3K/Akt pathway. 

**Figure 4:**
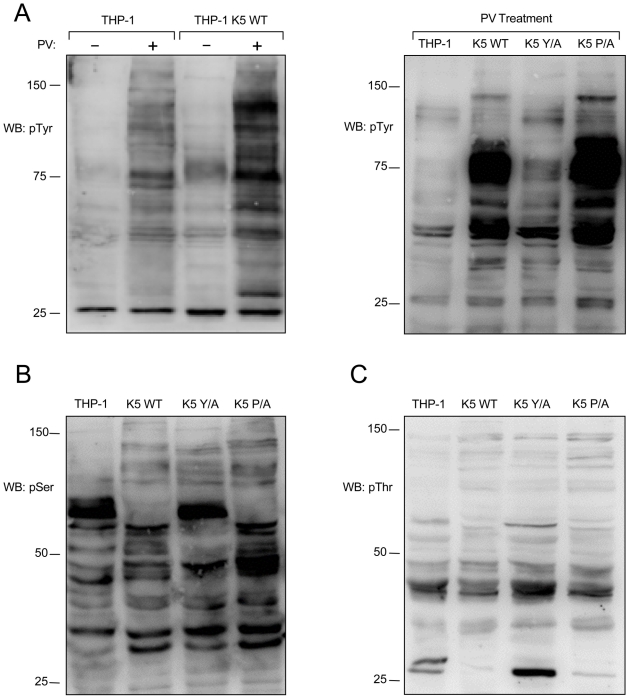
K5 alters tyrosine, serine and threonine phosphorylation. (**A, left panel**) Vector- and K5 WT-expressing THP-1 cells were mock (-) or pervanadate (PV) treated for ten minutes and normalized whole cell lysates were subjected to western blot (WB) using an anti-phospho-tyrosine (4G10) (pTyr) antibody. Normalized whole cell lysates from PV treated THP-1 cell lines, as indicated, were subjected to western blot using (**A, right panel**) anti-phospho-tyrosine (4G10) (pTyr), (**B**) anti-phospho-serine (pSer) or (**C**) anti-phospho-threonine (pThr) antibodies. Data for all panels are representative of three independent experiments.

To check the activation of RTKs and proximal signaling, we first examined total tyrosine phosphorylation. Normalized WCL of vector- and WT K5-THP-1 cells, mock or pervanadate (PV) treated for ten minutes, were subject to WB with a p-Tyr antibody. In these experiments, the WT K5-THP-1 cells contained increased amounts of total p-Tyr reactive proteins, as compared with vector-expressing THP-1 cells, but the pattern of phosphorylation between the two cell lines looked largely similar (Fig. 4A, left panel). The amount and pattern of phosphorylation of the K5 Y/A- and K5 P/A-expressing THP-1 resembled those of vector- and WT K5-THP-1 cells, respectively (Fig. 4A, right panel).

Normalized WCL of the THP-1 lines were next subjected to WB with either p-Thr or p-Ser antibodies. Both the WT K5- and K5 P/A-expressing THP-1 cells had alterations in serine and threonine phosphorylation, as compared with vector- or K5 P/A-expressing THP-1, indicating downstream signaling activity is also altered (Fig. 4B and C).

### K5-expressing THP-1 activate a specific cluster of RTKs

Since there were clear increases in tyrosine phosphorylation and altered overall phosphorylation in WT K5-expressing THP-1 cells, we next examined whether RTKs are differentially activated in vector- and K5-expressing THP-1. Following overnight serum starvation and treatment for 10 min with 10% FBS, normalized WCL of vector- or WT K5-expressing THP-1 cells were incubated with an array spotted with antibodies against 42 different RTKs. Activation of captured RTKs was then interrogated using a p-Tyr antibody. Multiple independent experiments demonstrated variable, but increased activation of Axl, Flt-3, Flt-4 and PDGFR-ß in WT K5-expressing THP-1 cells, as compared with vector-expressing cells. Interestingly, vector-THP-1 cells demonstrated activation of both the MCSF-R and Insulin-R that was not seen in the K5-expressing cells (Fig. S2).

### RTK inhibitors reduce growth rate of K5-expressing THP-1

**Figure 5:**
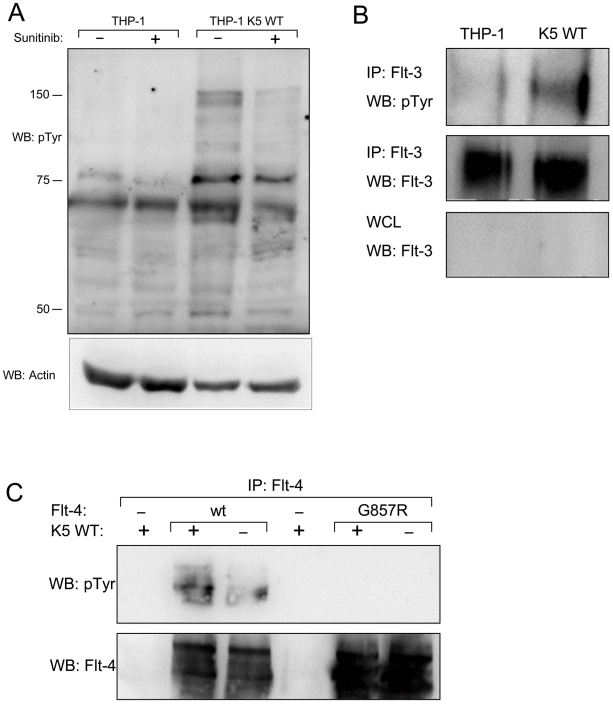
THP-1 cells stably expressing K5 WT have sunitinib-sensitive increased RTK phosphorylation. (**A**) Normalized whole cell lysates from THP-1 cells, mock (PBS) or sunitinib (2 µM) treated, were subjected to western blot using an anti-phospho-tyrosine (4G10) (pTyr) antibody (top panel) and reprobed with an anti-actin antibody (bottom panel) to demonstrate equal loading. (**B**) Normalized WCL from parental or K5 WT-expressing THP-1 cells were subject to immunoprecipitation (IP) using anti-Flt-3 antibodies followed by western blot (WB) using an anti-phospho-tyrosine (4G10) (pTyr) antibody (top panel). The IP (middle panel)and whole cell lysates (WCL) (bottom panel) were probed with anti-Flt-3 antibodies, as controls. (**C**) 293T cells were co-transfected with expression constructs for K5 and Flt-4 or a Flt-4 mutant (G857R). Two days post-transfection, cell lysates were subjected to IP using anti-Flt-4 antibodies. western blot (WB) was performed using anti-phospho-tyrosine (4G10) (pTyr) antibodies (top panel) and re-probed with anti-Flt-4 antibodies (bottom panel). Data for all panels are representative of three independent experiments.

As a first verification of the array data, cells were serum starved overnight and then treated with a relatively plieotropic RTK inhibitor, sunitinib (2µM), or DMSO, followed by stimulation with 10% FBS for ten minutes. Equal amounts of WCL were subjected to WB with a p-Tyr antibody. While there was only low level phosphorylation in vector-expressing THP-1 cells, either or without sunitinib treatment, WT K5-THP-1 cells demonstrated robust phosphorylation that was largely abrogated by sunitinib (Fig. 5A). Examination of the impact of sunitinib on cell growth produced less clear results. Incubation of vector- or K5-expressing THP-1 with 2µM sunitinib in 2%FBS resulted in a nearly equivalent average increase in doubling time, 1.33 fold (28.8 hrs to 38.4 hrs) and 2.06 fold (21.3 hrs to 39.6 hrs), respectively (data not shown). Interestingly, however, we also observed that treatment with sunitinib blocked acidification of the medium and reduced lactate production on a per cell basis (data not shown).

As an additional validation of the RTK array data, identified receptors were examined individually in THP-1 cell lines. First, normalized WCL of the THP-1 lines, treated with PV and 10% FBS for ten minutes post overnight serum starvation, were subjected to immunoprecipitation (IP) using a Flt-3 antibody followed by WB with a p-Tyr antibody. There was increased tyrosine phosphorylation of Flt-3 in WT K5- compared to vector-expressing THP-1 cells, while total Flt-3 levels remained the same (Fig. 5B). Similar results were seen for PDGFR-ß and Axl (data not shown). Due to experimental limitations, the activation of Flt-4 was examined in HEK-293T cells by transiently expressing K5 and either Flt-4 or a Flt-4 protein containing a mutation in the kinase domain, which renders it inactive. Following transfection, WCL were subjected to IP with a Flt-4 antibody followed by WB with a p-Tyr antibody. The phosphorylation of Flt-4 was increased the presence of K5, as compared to vector (Fig. 5C). As expected, this phosphorylation was not seen for Flt-4 containing a mutation in the kinase domain.

### K5-expressing THP-1 cells are more responsive to Flt-3L and PDGF-ß

**Figure 6:**
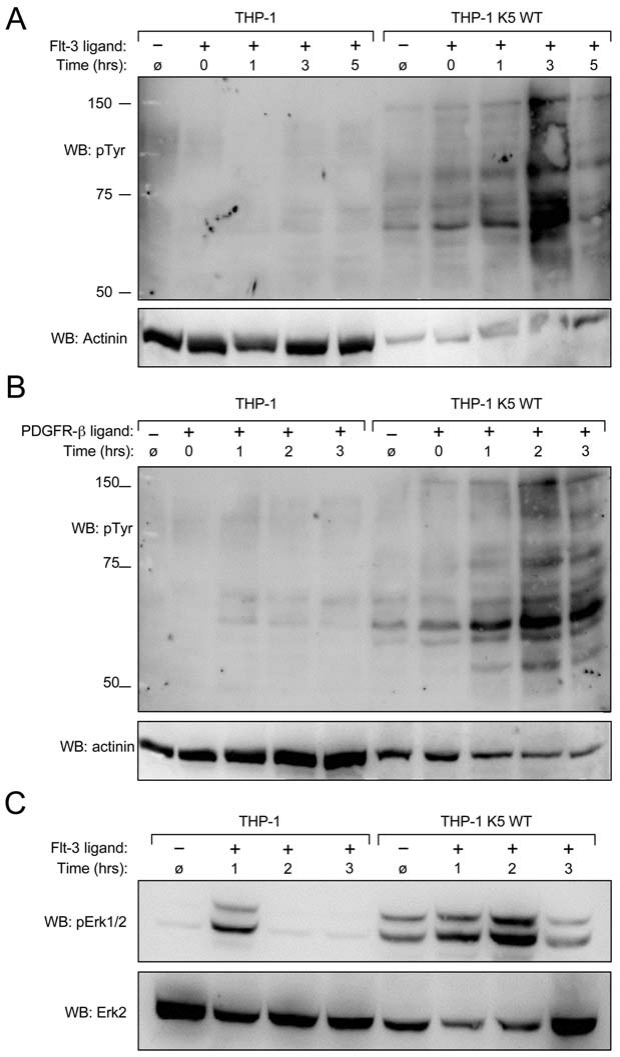
K5 WT-THP-1 cells are more responsive to Flt-3 and PDGF-ß-induced signaling and have increased Erk activation. Equal numbers of vector- and K5 WT-expressing THP-1 cells were serum starved overnight and stimulated with (**A and C**) Flt-3L (40 ng/ml) or (**B**) PDGF-ß (20 ng/ml) for ten minutes after which cells were washed and then treated with pervanadate for ten minutes at the times indicated. Western blot (WB) was performed on normalized whole cell lysates using (**A and B**) anti-phospho-tyrosine (4G10) (pTyr) or (**C**) anti-phospho-Erk antibodies. For panels **A and B**, 10 ug of THP-1 K5 WT lysate and 30 ug of THP-1 lysate were loaded to make comparison of bands easier between the two cell lines. Equal amounts of each lysate, 30 ug/lane, were loaded for panel **C**. Blots were re-probed for actinin (**A** and **B**) or total Erk (**C**). Data are representative of three independent experiments.

Given the increased phosphorylation of several RTKs in K5-expressing THP-1, combined with the increased doubling time in the presence of low serum concentrations, we next explored whether K5 was acting not to directly activate these receptors, but to modulate their responses to ligands. Equal numbers of cells from each of the THP-1 lines were serum starved overnight and then were Flt-3 ligand or mock treated for ten minutes followed by washing and incubation at 37°C for various times, as indicated. Normalized WCL from PV treated cells were subjected to WB with a p-Tyr antibody. As shown in Figure 6A, WT K5-expressing THP-1 cells are comparatively more responsive to Flt-3 ligand, with the most robust signaling activity seen around 3 hours post stimulation, while there was a minimal, but detectable response in vector-expressing cells. Similar results were obtained when the experiment was repeated with PDGF-ß (Fig. 6B).

To determine whether activation of the RTKs by specific ligands induced downstream MAPK signaling, we focused on the activation of Erk in the THP-1 cell lines. A similar time course experiment was set up as for the Flt-3 activation experiment, but the WCL were probed with p-Erk1/2 and Erk2 antibodies after blotting. Erk1/2 demonstrated a higher basal and a longer duration of activation in WT K5-expressing THP-1 cells, as compared to vector-THP-1 cells (Fig. 6C). Similar results were obtained following PDGF-ß stimulation (data not shown).

### K5-expressing THP-1 cells have lower surface, but increased intracellular RTK levels

**Figure 7:**
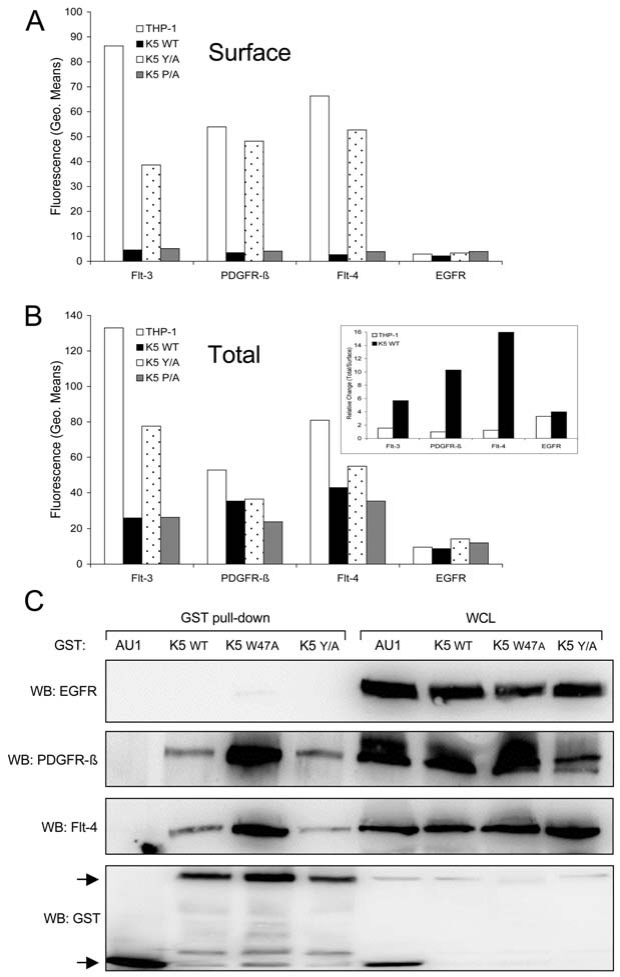
K5 interacts with and alters RTK localization. Equal cell numbers of the indicated THP-1 lines were fixed with paraformaldehyde (PFA) and (**A**) stained without permeabilization to determine surface expression or (**B**) permeabilized with saponin prior to staining to determine total expression of Flt-4, PDGFR-ß, Flt-3 and EGFR by flow cytometry. Data are representative of three independent experiments. (**B, Inset**) The relative ratio of surface versus total RTKs was determined for vector- and K5 WT-expressing THP-1 cells. (**C**) 293T cells were co-transfected with expression constructs for EGFR, PDGFR-ß, or Flt-4 and the indicated GST expression constructs. After two days, lysates were subjected to GST pull-down using glutathione-sepharose beads. Purified proteins and whole cell lysates (WCL) were subjected to western blot (WB) using anti-EGFR, -Flt-4 or -PDGFR-ß antibodies, followed by re-probing with anti-GST antibodies. Arrows indicate GST or GST-K5 WT and mutant specific bands. Data are representative of three independent experiments.

Since there is increased signaling through selected RTKs in WT K5-expressing THP-1 cells, we first hypothesized that K5 prevented RTKs from being internalized and degraded, thus allowing for a longer duration of signaling from the cell surface. Contrary to our hypothesis, examination of the THP-1 lines by flow cytometry revealed that while vector- and K5 Y/A-expressing THP-1 lines had a high surface expression of all three RTKs, WT K5- and K5-P/A-expressing lines had minimal expression (Fig. 7A). As expected, both the K5 W47A- and K5 mZn-expressing THP-1 showed surface levels of all three RTK comparable to vector-expressing cells (data not shown). Examination of the EGFR, which was present in our phospho-RTK array analysis but did not show differential activation in the K5-expressing cells, demonstrated no difference in cell surface expression for any of the lines. We next examined whether there were increases in intracellular RTK expression. Flow cytometric analysis of permeabilized cells revealed that while the relative ratio of total to surface RTK amounts in vector- and K5 Y/A THP-1 cell lines was approximately 1, this ratio was considerably greater in both the WT K5- and K5 P/A-expressing THP-1 cell lines (Fig. 7B and inset). Again, no effect of K5 WT or K5 P/A was observed on EGFR with regards to total protein or the ratio of surface to intracellular protein.

### K5 forms complexes with the activated RTKs

Given the increased amounts of internalized RTKs in WT K5-expressing THP-1 cells, we next examined whether K5 was able to form complexes with the identified RTKs. HEK-293T cells were co-transfected with either GST-fusion constructs of K5 along with EGFR (negative control), PDGFR-ß, or Flt-4 constructs. After pulldown with glutathione beads, WB was performed on precipitated proteins and WCL using EGFR, Flt-4 and PDGFR-ß antibodies. Neither the WT K5 or K5 mutant constructs were able to interact with EGFR, although expression levels were equivalent (Fig. 7C). All of the GST-tagged K5 constructs were able to interact with both PDGFR-ß and Flt-4, while no binding was seen with the empty vector expressing only the GST-AU1 fusion construct. Interestingly, K5 Y/A, which has been shown to be deficient in mediating endocytosis of other K5 targets, can still interact with the RTKs suggesting that simple interaction with the RTKs is not sufficient for increased signaling [31]. Additionally, increased association of both PDGFR-ß and Flt-4 were seen with the K5 W47A construct. This mutant of K5 lacks E3 ubiquitin ligase activity, indicating this function is not necessary for the interaction (data not shown).

### The endocytosis of RTKs is necessary for increased signaling in presence of K5

**Figure 8:**
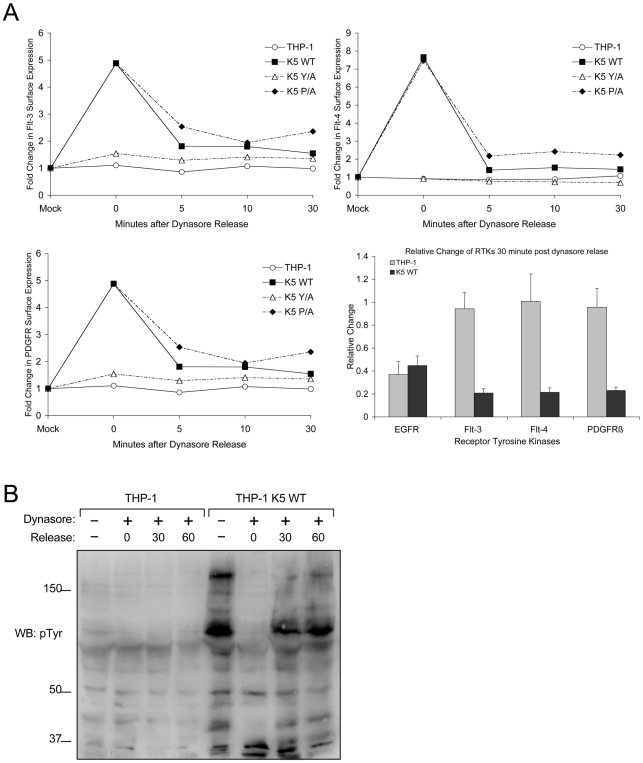
K5 mediates rapid internalization of RTKs from the surface leading to increased signaling. (**A**) Equal numbers of the indicated THP-1 lines were mock or Dynasore (80 µM) treated for an hour at 37°C. Cells were then released at 37°C in serum free media for 0, 5, 10 or 30 minutes. At the indicated time points, cell aliquots were fixed and stained for surface levels of Flt-3, Flt-4 and PDGFR-ß followed by flow cytometric analysis. Representative experiments for each of the three receptors demonstrating the relative change in the expression of each RTK normalized to the fluorescence (geometric means) of mock treated samples is shown as line graphs, while the overall endocytosis rate of the receptor tyrosine kinases over the 30 minutes following dynasore release normalized to the time 0 point is shown as a bar graph with standard deviations. Line graph results are representative of at least three experiments (**B**) Equal numbers of vector- and K5 wt-expressing THP-1 were seeded in media containing 2% FBS. Following treatment with DMSO (mock) or Dynasore (80 µM) for 60 min and release in Dynasore-free medium at 37°C for 0, 30 or 60 minutes normalized whole cell lysates were prepared and subjected to western blot (WB) with an anti-phospho-tyrosine (4G10) (pTyr) antibody.

To further determine if K5 mediates endocytosis of RTKs from the surface, we did a time course study in the THP-1 lines with dynasore, a reversible inhibitor of dynamin [51], [52]. Following treatment for one hour, drug was washed out and cells were placed at 37°C for various amounts of time, after which cells were stained and analyzed by flow cytometry. While the surface expression of RTKs in vector-THP-1 cells changed minimally pre- and post-dynasore treatment, the surface expression of Flt-3, Flt-4 and PDGFR-ß in K5-THP-1 cells went up 5 fold, 5.5 fold and 8 fold, respectively (Fig. 8A). These increases were mirrored in the K5 P/A-expressing cells, while the K5 Y/A-expressing cells resembled vector-expressing THP-1. These surface levels in the K5 WT- and K5 P/A-expressing cells decreased rapidly following drug removal to the levels seen in mock treated cells.

Given that altered signaling pathway activation was seen only in cells expressing K5 variants capable of inducing RTK endocytosis, we next explored whether endocytosis was required for increased signaling. Equal numbers of THP-1 cells were either mock or dynasore treated for 60 min., followed by washing and re-suspension in medium containing 10% FBS. Cells were collected pre-dynasore treatment, after 1 hour of treatment and at two time points post-washout. WCL from each time period were subjected to WB with a p-Tyr antibody. K5-expressing THP-1 cells treated with dynasore had markedly decreased tyrosine phosphorylation when compared to mock (DMSO) treated cells, while there was minimal change in vector-expressing THP-1 cells with or without dynasore treatment (Fig. 8B). Following dynasore removal we observed a rapid recovery of tyrosine phosphorylation in the K5-expressing THP-1 cells. Interestingly, increased p-Tyr reactivity was not seen to increase if cells were re-suspended in serum-free medium following drug washout (data not shown).

## Discussion

In this study, we show for the first time that a viral E3 ubiquitin ligase, KSHV K5, can act as a pro-oncogenic factor altering metabolism and increasing pro-growth, proliferation and survival signaling cascades. K5-expressing monocyte cell lines display increased dependency on glucose and glutamine, coupled with higher rates of glucose uptake and lactate production (Figs. 2B, C & D, Fig. S1B). TIME cells stably expressing K5 also display many of these changes suggesting that this viral protein might be acting to alter metabolism both in the spindle cells making up the bulk of the tumor, in addition to the surrounding stroma and inflammatory cells and we are actively investigating this possibility (Figs. 2D & 4E). More importantly, the discovery of a tumor virus protein capable of inducing the Warburg effect provides the basis for a model system to look more deeply at the role of metabolic dysfunction in cancer.

We have shown that K5 is able to extend the duration and increase the sensitivity of receptor tyrosine kinase signaling (Fig. 6). Given that signaling from these receptors is still ligand dependent and enhanced proliferation of K5-expressing THP-1 cell lines is serum dependent, we do not believe that K5 is inducing receptor activation in the absence of these serum factors (Figs. 1B & 8B). However, this modulation by K5 results in a longer time course of activation of multiple signaling cascades at a lower concentration of ligand, including Akt and MAPK, leading to subsequent increases in aerobic glycolysis and proliferation. KSHV infection of endothelial cells has been shown by others to increase HIF protein levels and confer increased survival through activation of the Akt/mTOR pathway signaling, but no mechanism of pathway activation was demonstrated [53], [54]. We are currently exploring the role that K5 plays in this upregulation of HIF-1α during infection using viruses deleted for K5.

The mechanisms by which K5 alters monocyte metabolism are likely to be complex. EGFR amplification in cancer has been implicated in stabilizing glucose transporters, independent of its ability to increase activation of down stream signaling pathways [55]. This ability to stabilize glucose transporters has not been examined for other RTKs, however our data shows increases in mRNA levels for GLUT4 and further, we have also observed a decrease in lactate production in K5-expressing THP-1 cells treated with sunitinib, suggesting that increased RTK signaling and not transporter stabilization is responsible for the alterations in metabolism (data not shown). We also observed that alongside their increased dependence on glucose, K5-expressing cells also displayed an increased dependence on glutamine with decreases in mitochondrial potential (Fig. 3B & Fig. S1B). Interestingly, other inhibitors including AG1295 (PDGFR-ß inhibitor, 50μM), Rapamycin (mTor inhibitor, 100nM) and Triciribine (AKT inhibitor, 1μM) also blocked medium acidification, while MEK and JNK inhibitors did not, demonstrating the utility of this system for examining Warburg induction and the linkage between glutaminolysis and mitochondrial biogenesis.

We have shown that changes in tyrosine phosphorylation correspond with the growth and metabolism phenotype. The vector- and Y/A- expressing cells, which do not display markers of the Warburg effect, have decreased tyrosine phosphorylation compared with wild-type K5- and P/A-expressing cells, suggesting that increased signaling is probably coupled with altered metabolism (Figs. 1, 2 & 4). This linkage has been observed previously in the case of fibroblast growth factor receptor, with constitutive signaling causing both increased phosphorylation and partial inhibition of pyruvate kinase M2, leading to decreased respiration [37]. The KSHV literature suggests that KS spindle cells, which form the bulk of the tumor, are more dependent on signals from growth factor signaling pathways, but do not contain activating mutations. This has lent support to the rationale of using sunitinib, a pleiotropic RTK inhibitor targeting a variety of RTKs including Flt-3, PDGFR-α/ß and VEGFRs, for the treatment of KSHV induced pathogenesis [56], [57]. Intriguingly, sunitinib inhibits K5-mediated activation of RTKs and decreases lactate production (Fig. 5A and data not shown). Additionally, K5 alteration of metabolism and proliferation is also sensitive to Rapamycin, another drug that is showing promise against KS in the clinic [58]-[61] (Fig. S3). The data presented in this manuscript open the possibility that these drugs, in addition to targeting signaling pathways in the infected spindle cells, might also be reducing the ability of infected cells to aid in tumor progression through the release of lactate.

The fact that endocytosis of the RTKs, not just interaction with K5, is required for the increased signaling, as evidenced by the ability of dynasore to block increased tyrosine phosphorylation and the interaction of K5 Y/A with the RTKs in pulldown assays, is a very interesting finding (Figs. 7C and 8B). There are a large number of mechanisms by which RTK signaling, without mutation of the RTK, can be dysregulated in cancer. Our model shows a requirement for an intact internalization machinery and argues that very subtle mutations in trafficking proteins, that do not increase cell surface receptor levels and in fact decrease surface levels, could lead to unregulated receptor signaling. In keeping with this model, it is thought that the full biological activity of RTKs is conferred only after endocytosis, activating signal transducers like Erk1/2 for robust and sustained signaling [62]. We have observed and are currently exploring whether K5 interaction with two different cellular proteins, CD2AP, a Cbl interacting protein and filamin, a component of the cytoskeletal machinery, could be important in this dysregulation (data not shown).

We have been unable thus far to observe increased ubiquitylation of any of the explored RTKs in THP-1 cells in the presence of K5. However, we did observe that while the K5 W47A mutant is able to interact with the RTKs in transient assays (Fig. 7C), it did not induce either increased endocytosis or changes in THP-1 proliferation (data not shown). Equally, the K5 mZn construct, which lacks a functional RING-CH domain, is also unable to modulate increased RTK endocytosis. Together, this data can be interpreted to mean that ubiquitylation is likely playing a role in the alteration of RTK signaling by K5. We are currently engineering a series of THP-1 cell lines to express HA-tagged ubiquitin to explore this possibility.

Numerous studies have documented a role for KSHV infection in regulating cytokine production by monocytes and macrophages [14]-[17], [63], [64]. *In toto*, these published studies suggest a mechanism whereby KSHV infection of monocytes, as well as endothelial cells, drives the production of pro-inflamatory cytokines promoting KS tumor outgrowth [65], [66]. Our data extend this hypothetical model further in two ways. First, our findings demonstrate that K5 can alter the responsiveness of expressing cells to growth factors, allowing for responsiveness to very low levels of these pro-growth mediators. Second our data provides additional evidence for the possibility that viral infection and K5 expression, in particular, could be driving changes in the tumor microenvironment. Published work from Delgado *et al.* has demonstrated, for example, that at early time points post-infection KSHV drives increased lactate production in TIME cells [35]. Stable K5 expression in TIME cells similarly led to increased lactate production, as well as alterations in PKM2 phosphorylation, opening the possibility that the increases in lactate production seen during infection are due to the early production of K5 protein (Fig. 2A, C & E). Lending weight to this possibility is the fact that we did not observe increased lactate production in KSHV-infected TIME cells when we looked at time points ranging from 4-8 weeks post-infection when K5 protein expression is low (data not shown). We are actively working to produce and characterize a K5 gene deleted virus to further pursue this question.

Overall, in this report we provide evidence that the KSHV K5 protein is able to drive alterations in cellular signaling cascades, potentially contributing to KSHV oncogenesis. We believe that this is the first report of a virally-encoded E3 ubiquitin ligase contributing to changes in cellular metabolism and proliferation through a direct alteration of RTK endocytosis rates and signaling output. These data open a new line of inquiry the mechanisms of KSHV oncogenesis and the biology of both K5 and its cellular homologues.

## Methods

### Cell culture, transfection and growth curves

THP-1 and 293T cells were grown in RPMI plus 10% Fetal Bovine Serum (FBS) and Dulbecco's modified Eagle's medium plus 10% FBS, respectively. TIME cells were grown in EBM medium with complete bullet kit (Lonza, Walkersville, MD). Stable cell lines were established by retroviral transduction, as described previously [32]. THP-1 cells were selected with 1 mg of G418 per ml for 2 weeks following transduction and then characterized for construct expression by radioactive IP. The 293T cell line was transfected using Transfectin (Bio-Rad) according to the manufacturer's recommendations. To determine growth rates, cells were seeded, unless otherwise indicated, at 1x10^5^/ml in RPMI supplemented with the indicated amount of FBS. Viable cell numbers were determined using Trypan Blue staining and counting by hematocytometer. For growth curves with sunitinib, either DMSO (Mallinckrodt Baker) or 0.75µM sunitinib (Sigma-Aldrich) was added to cultures and replenished daily.

### Plasmid constructs

All pEF-K5 WT and mutant constructs used in this paper were generated using oligonucleotide-directed mutagenesis described previously [31]. Each of the K5 constructs were transfered into pLXSN (gift of Dr. D. DiMaio) for retroviral transduction or pcDEF-GST-AU1 (gift of Dr. J. Jung) for pulldown experiments and sequence verified. The Flt4, EGFR and PDGFR-ß expression constructs were the gifts of Drs J. Groopman, J. Jung and D. DiMaio, respectively.

### Antibodies

#### Antibodies for flow cytometry

From Santa Cruz Biotechnology: anti-Flt-3/Flk-2 (SF1.340), anti-Flt-4 (C-20), anti-PDGFR-ß (958); From Dako: MHC class I (clone W6/32); From Becton-Dickinson: fluorescein isothiocyanate-, PE-, or allophycocyanin-conjugatedisotype controls (Simultest), all secondaries.

#### Antibodies for IP

From Santa Cruz Biotechnology: anti-Flt-3 (SF1.340), anti-Flt-4 (C20), anti-PDGFR-ß (958); From Millipore: anti-EGFR (rabbit monoclonal).

#### Antibodies for WB

From Santa Cruz Biotechnology: anti-HIF-1α (H-206), anti-p-Akt1/2/3(Ser 473)-R, anti-Akt1/2 (N-19), anti-p-thr (BDI141), anti-Flt-3/Flk-2 (SF1.340), anti-Flt-4 (C-20), anti-PDGFR-ß (958), anti-α-actinin (C-20), anti-Erk2 (D-2) or anti-GST (B-14); From Millipore: anti-phosphotyrosine (4G10 Platinum), anti-EGFR (04-338), anti-p-Akt1 (Thr 308); From Abcam: anti-phosphoserine (ab9332-100); From Cell Signalling Technology: anti-phospho-p44/42 MAPK (Erk1/2) (Thr202/Tyr204) (D13.14.4E).

### Cell cycle and apoptosis assays

THP-1 cells lines were seeded in triplicates, 5 x 10^5^ per sample, in the indicated amount of serum. Five days post seeding they were stained for 15 minutes using Annexin-V-FLOUS staining kit (Roche), according to the manufacturer's recommendations and analyzed by flow cytometry. Cell cycle analysis was performed by standard propidium iodide (Sigma) staining protocol followed by flow cytometry and analysis with FlowJo software (Tree Star).

### Measurement of lactate production, glucose uptake and mitochondrial function

To measure lactate production, cells were seeded at 1x10^5^ cells/ml and were enumerated and supernatant was taken at the indicated time points. Lactate concentration in the supernatants was measured by colorometric assay (Biovision) according to the manufacturer's recommendations. For glucose uptake assay, 5x10^5^ cells per sample were washed and starved in PBS for 15 minutes then incubated with 0.05mM 2-NBDG (Invitrogen) plus 5mM Glucose-D (Mallinckrodt Baker) in PBS for a period of 30 minutes, followed by flow cytometric analysis. Mitochondrial function was determined by staining with Mitotracker Green and Mitotracker Red (Invitrogen) for 30 minutes, according to the manufacturer's recommendations.

### Receptor levels and endocytosis

Cells at 5x10^5^ per sample were fixed with 3% paraformaldehyde and incubated with indicated antibodies for 45 minutes in either flow buffer (0.5% FBS-PBS) or 0.2µM Saponin buffer for surface or total levels of RTKs, respectively. RTK internalization kinetics was determined by incubating cells, 6x10^6^ cells/sample, with DMSO (mock) or 80µM Dynasore (EMD Chemicals) for 1hr at 37°C. Cells were then washed and incubated at 37°C for the indicated time periods. At the indicated times, 0.5 x 10^6^ cells were transferred to ice and sodium azide was added to a final concentration of 0.05% to stop endocytosis. Following collection of all time points, cells were then fixed and stained with the indicated RTK antibodies.

### Immunoprecipitation, GST pulldown and immunoblot assays

Proteins were extracted from cells, normalized by BCA assay (Pierce) and subjected to adsorption with protein A-agarose (SCBT) plus antibody for IP, incubation with glutathione-sepharose agarose (Amersham) for pulldown, or directly subjected to SDS-polyacrylamide gel electrophoresis and WB, as previously described [31]. The membranes were incubated with appropriate secondary antibody conjugated with horseradish peroxidase (SCBT) and were visualized using the enhanced chemiluminescence system (Roche Diagnostics) and a LAS 3000 camera (FujiFilm).

### Human Phospho-RTK arrays

To examine receptor activation, THP-1 cells were serum starved overnight and stimulated with 10% FBS and pervanadate for 10 minutes. Normalized WCL from cells were incubated with a Human Phospho-RTK Array (R& D systems) and developed according to the manufacturer's recommendations.

### RT-Profiler PCR Array

RNA was extracted from 1*10^6^ vector- or K5-expressing THP-1 cell lines, grown in RPMI + 10% FBS, using the RNeasy Mini Kit (Qiagen). The Human Hypoxia Signaling Pathway RT-Profiler PCR Array (SABiosciences) was performed according to the manufacturer's protocols.

### Ligand-stimulation assay

For ligand stimulation studies, THP-1 cells were serum starved overnight and stimulated with 40ng/ml Flt-3/Flk-2 ligand (Calbiochem) or 20ng/ml PDGF-ß (Millipore) for ten minutes. After ligand washing, the cells were resuspended in serum free RPMI media and aliquot of cells were collected at the indicated time points and subjected to WB, as described above.

## Supporting Information

Figure S1 K5-expressing THP-1 are insensitive to charcoal depletion of serum factors and display greater glutamine dependancy.
(0.32 MB EPS)

Figure S2 K5-expressing THP-1 cells display altered receptor tyrosine kinase activation.
(0.43 MB EPS)

Figure S3 Growth of K5-expressing THP-1 cells is sensitive to rapamycin.
(0.21 MB EPS)

Table S1 qRT results of hypoxia-related genes altered in KSHV K5-expressing monocytes.
(2.87 MB EPS)
